# Supplementary material for: MetaRibo-Seq measures translation in microbiomes
Source: Nat Commun. 2020 Jun 29;11:3268. doi: 10.1038/s41467-020-17081-z (PMC7324362; doi:10.1038/s41467-020-17081-z)
Supplement: Supplementary file 10 — Supplementary Data 7 [file 41467_2020_17081_MOESM10_ESM.zip › File2/Confidence_VeryHigh_Taxonomy/175498_out.krona.html]

Javascript must be enabled to view this page.

members
magnitude
magnitudeUnassigned
count
unassigned
taxon
rank

175498\_out

4

2
4
superkingdom

1239
phylum
4

1
class
526524

526525
order
1

128827
family
1

1
genus
1470349


SRS149879\_contig\_number\_contig-100\_101.155082
1574263
1
species

186801
3
class

order
3
186802

family
3
541000

species
2
1898205

SRS014235\_contig\_number\_26487SRS018575\_contig\_number\_3800

216851
1
genus

species
1

SRS013476\_contig\_number\_contig-100\_4663.185185
1262897
